# SPACE for physical activity - a multicomponent intervention study: study design and baseline findings from a cluster randomized controlled trial

**DOI:** 10.1186/1471-2458-11-777

**Published:** 2011-10-10

**Authors:** Mette Toftager, Lars B Christiansen, Peter L Kristensen, Jens Troelsen

**Affiliations:** 1 Institute of Sports Science and Clinical Biomechanics, University of Southern Denmark, Campusvej 55, 5230 Odense M, Denmark

## Abstract

**Background:**

The aim of the School site, Play Spot, Active transport, Club fitness and Environment (SPACE) Study was to develop, document, and assess a comprehensive intervention in local school districts that promote everyday physical activity (PA) among 11-15-year-old adolescents. The study is based on a social ecological framework, and is designed to implement organizational and structural changes in the physical environment.

**Methods/design:**

The SPACE Study used a cluster randomized controlled study design. Twenty-one eligible schools in the Region of Southern Denmark were matched and randomized in seven pairs according to eight matching variables summarized in an audit tool (crow-fly distance from residence to school for 5-6^th ^graders; area household income; area education level; area ethnicity distribution; school district urbanity; condition and characteristics of school outdoor areas; school health policy; and active transport in the local area). Baseline measurements with accelerometers, questionnaires, diaries, and physical fitness tests were obtained in Spring 2010 in 5-6^th ^grade in 7 intervention and 7 control schools, with follow-up measurements to be taken in Spring 2012 in 7-8^th ^grade. The primary outcome measure is objective average daily physical activity and will be supported by analyses of time spent in moderate to vigorous activity and time spent sedentary. Other secondary outcome measures will be obtained, such as, overweight, physical fitness, active commuting to/from school and physical activity in recess periods.

**Discussion:**

A total of 1348 adolescents in 5-6^th ^grade in the Region of Southern Denmark participated at baseline (n = 14 schools). The response rate was high in all type of measurements (72.6-97.4%). There were no significant differences between intervention and control groups at baseline according to selected background variables and outcome measures: gender (p = .54), age (p = .17), BMI (p = .59), waist circumference (p = .17), physical fitness (p = .93), and physical activity (accelerometer) (p = .09).

The randomization and matched pair design produced equivalent groups according to central outcome measures and background variables. The SPACE for physical activity Study will provide new insights on the effectiveness of multicomponent interventions to improve adolescents' physical activity level.

**Trial registration:**

Current Controlled Trials ISRCTN79122411

## Background

The physical, mental, and social health benefits of physical activity (PA) in children and adolescents are well documented [1-4]. Growing evidence suggests that PA in childhood and adolescence will track into adulthood and can prevent lifestyle related diseases such as cardiovascular diseases, some cancers, osteoporosis and diabetes that are manifested later in adulthood [2,4-7]. Despite the benefits of PA, a significant number of young people in Denmark and other Western countries do not reach recommended levels of PA [8,9].

Efforts to increase levels of PA in children and adolescents have primarily relied on community, family or school settings, and individual or educational approaches, but results have been mixed [10-13]. As young people tend to spend a large proportion of their waking hours at school, schools have long been recognised as potentially effective settings for public health initiatives, and PA interventions and can largely benefit the most socially disadvantaged groups of children [11,14-16].

In a systematic review from 2007 [10] based on 33 intervention studies in children and 24 studies in adolescents(12-18 y), it was concluded that among studies conducted in children, limited and inconclusive evidence for an effect was found. On the other hand, in adolescents, school based interventions involving family, community, and/or multicomponent interventions can increase physical activity in adolescents. However, these conclusions were based on few studies. In the review, out of the 24 studies in adolescents, six of them evaluated a multicomponent intervention, all in the school setting, and only three of these studies were large high quality randomized trials, all showing positive results. Most studies reviewed were conducted in USA, and only a few in a European context. It is concluded that in general there is a need for more systematic and high quality studies [10].

The effect of multicomponent interventions is in keeping with the ecological approach to behaviour change, which states that as health behaviour is influenced at multiple levels, so should interventions to maximize effectiveness. One principle of ecological models is that they need to be tailored to specific behaviours, and has been identified as a suitable conceptual model for the design of PA interventions [10,17-20]. In the past decades, there has been an increasing interest in, and application of, ecological models in research and practice, due in part to the potential in guiding comprehensive approaches to change behaviour [21-23].

The multicomponent intervention study SPACE for physical activity (School site, Play Spot, Active transport, Club fitness and Environment) has been developed based on a social ecological framework. The SPACE study is designed to develop, document and assess a comprehensive intervention in local school districts to promote everyday PA among adolescents. The target group is students in 5-6^th ^grade (11-13 years of age) followed for two years, as several studies show a drastically decrease in the level of PA at the transition to teenage years [24-27]. In the SPACE Study we aim to reduce this age-related decline in PA level.

The research hypothesis to be tested is that a multicomponent PA promotion programme comprising different organizational and physical environmental intervention components can be designed and successfully implemented in collaboration with schools and municipalities in Denmark, and that the intervention will lead to increased PA levels among adolescents.

The purpose of this paper is to present the study design, the design of the intervention components, the data collection procedures, outcome measures and selected baseline characteristics of the SPACE study.

The SPACE study is registered in the Current Controlled Trials (ISRCTN79122411), and findings will be reported in accordance with the guidelines of the CONSORT statement [28].

## Methods/Design

### Study design

**Figure 1:**
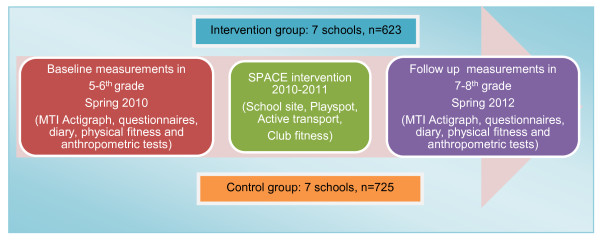
**Illustration of the SPACE study design**.

This study employed a cluster randomized controlled study design with baseline measurements in Spring 2010 (5-6^th ^grade) and follow-up to be collected in Spring 2012 (7-8^th ^grade) (Figure 1).

#### Estimation of sample size - power calculations

Sample size calculations were performed prior to the study. Conventional levels of statistical power (0.8) and level of significance (0.05) were used in the two-sided test The minimum detectable effect size between groups was determined at 60 counts/min, representing an approximate 10% difference in the outcome measure at follow-up. We used data from the Danish part of the European Youth Heart Study (EYHS) [29] to estimate the variability in the change in physical activity from baseline to follow-up. Data from the EYHS was also used to estimate the between school variation in physical activity in order to control for clustering within schools (ICC = 0.011). The between school variation was estimated while controlling for most of the school matching variables described below. All analyses were performed with statistical software STATA v10 using the modules Sampsi and Sampclus. The calculations showed that a minimum of 12 schools (6 control and 6 intervention schools) were required, based on an average number of 100 students per school.

#### Recruitment and randomisation

Denmark is divided into five regions with approximately 20 municipalities in each region. The municipalities are responsible for health promotion and school education at the primary level (kindergarten to 10^th ^grade). The main responsibility of the regions is to provide health care.

**Figure 2:**
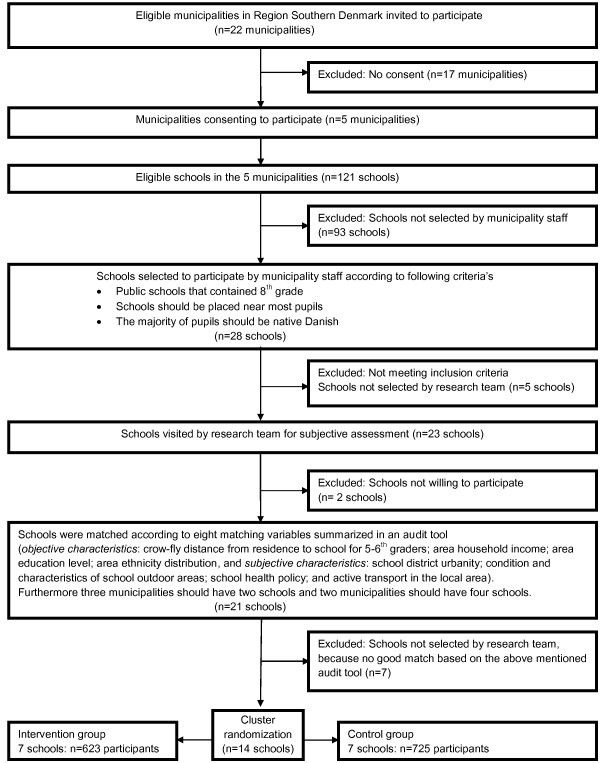
**Flow diagram of recruitment and randomization of schools in the SPACE for physical activity study**.

In spring 2009, all municipalities in the Region of Southern Denmark were invited to participate in the SPACE study. Five municipalities (Esbjerg, Nordfyn, Varde, Vejle and Sønderborg) out of 22 accepted the invitation and were asked to enroll public schools that contained 8^th ^grade. A total of 28 schools were recommended or deemed eligible by the municipalities, however, schools were considered ineligible for the study based on the following criteria: a) if placed in the countryside with more than 25% of all students living further than 2 km crow fly distance from the school; and b) if the majority of all students were non native Danish. Five schools were excluded by the research team because they did not meet the above mentioned criteria. This resulted in an enrollment of 23 schools in the five municipalities. To reduce the number of schools required according to the sample size estimation, we developed and used an audit tool. The audit tool consisted of a total of 8 school characteristics consisting of 4 objective and 4 subjective qualities from the 23 schools. The objective characteristics were: 1) crow-fly distance from residence to school for 5-6^th ^graders, 2) area household income, 3) area education level, and 4) area ethnicity distribution. The information was obtained from Statistics Denmark and with the use of Geographic Information System (GIS). The subjective information, which was based on interviews with municipality consultants and managing school personnel at each school site, consisted of the following parameters: 1) school district urbanity, 2) condition and characteristics of school outdoor areas, 3) school health policy, and 4) active transport in the local area. After visits at the school sites, 2 schools declined to participate in the project resulting in 21 eligible schools. A Spearman rank correlation analysis was conducted with the standardized values of the eight measures between the 21 schools. As part of the selection process, it was a given condition that Esbjerg and Varde municipalities participated in the project with four schools each, and the municipalities of Nordfyn, Vejle and Sønderborg participated with two schools each. The seven best matched pairs were selected for randomization, resulting in a total of 14 schools participating in the study. The schools were then randomized in an intervention group and a control group. Based on the eight school characteristics and the limited number of schools, a matched pair design was applied to optimize the randomization [30]. In order to increase power however an unmatched analysis of the matched data was adopted [30]. A flowchart illustrating the recruitment and randomisation can be seen in Figure 2.

Parents of the participating children received a passive informed consent form that explained the nature and procedures of the study. If parents and/or their child(ren) did not want to participate, they could withdraw during any stage. The study has been reviewed by the Danish Ethical Committee and they concluded that formal ethics approval was not required. The study is registered and listed in the Danish Data Protection Agency (reference number: 2009-41-3628).

### Intervention components

**Figure 3:**
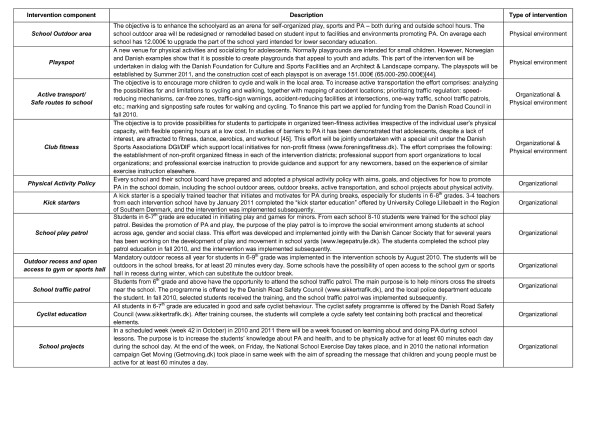
**Intervention components in the SPACE for physical activity study**.

The multicomponent intervention comprises four main areas: 1) the school's outdoor areas, 2) playspots 3) active transport and 4) club fitness. A total package of 11 intervention components combining both organizational and physical environment changes will be implemented in all 7 intervention schools. An overview of the 11 intervention components can be seen in Figure 3. A detailed description of the 11 intervention components can be seen at: http://www.forebyggelsescenter.dk.

### Implementation, organizing and anchoring

The development of the study design and the implementation of the intervention are carried out in close partnerships between the research team at University of Southern Denmark, the Region of Southern Denmark, the five municipalities, and the 14 schools. The Region of Southern Denmark serves as coordinator in relation to the municipalities and participates with process management. One fulltime employee is allocated by the region for this task. The municipalities involved are expected to finance intervention costs with support from various funds and a halftime employee is available for the development and coordination of the local interventions. The schools, which are the targets of the interventions, performed the tasks of specifying, adapting and implementing the interventions. Thus, a primary aspect of the intervention was to involve school teachers, local institutions and organizations in anchoring the initiatives. It is however, important to establish that the overall content of the 11 intervention components were defined prior to the project start, and are the same in all 7 intervention schools. In Denmark the conditions for collaboration between universities and municipalities are present, since the municipalities have taken on the responsibility for a number of health promotion and disease prevention tasks following a comprehensive structural reform in 2007. Thus, the original initiative to develop the project originates from collaboration between Region of South Denmark and municipalities in the region. Furthermore, the proposed collaborative structure is already familiar to the partners.

### Data collection procedures

The evaluation of the SPACE study is split into three main parts: 1) quantitative evaluation of physical activity, physical fitness, and determinants for physical activity, 2) anthropologic evaluation of the intervention effect in two schools, and 3) process and health economic evaluations of the intervention. This study protocol focuses on the first part that consists of objective assessment of PA by accelerometry measures, physical fitness and anthropometric tests, questionnaires and activity diaries (table 1). Below is a thorough description of the quantitative methods of measurements used in the study.

**Table 1:** Description of baseline measurements April-June 2010, study population (adolescents: n = 1348, parents: n = 1348, school managements: n = 14), and response rate

*Measurement*	*Who*	*What*	*When*	*Response rate*
Questionnaire	Adolescents	Students filled out an e-survey in a school session with questions about physical activity, health, neighbourhood, school etc.	Duration: 30 minutes. During the week when wearing accelerometer in April-June 2010	97.4% (n = 1313)

Diaries	Adolescents	Every morning during one week the students filled out a diary with information about active transportation, and use of accelerometer	5 minutes each school day in 1 week (5 weekdays and 2 weekend days) simultaneously with wearing the accelerometer, in April-June 2010	96.6% (n = 1302)

Physical fitness and anthropometric tests	Adolescents	Students (without health problems) participated in a physical testing session of 11/2 hour including: body measurements (weight, height, waist circumference), running test, agility test, fitness test	Duration 11/2 hour, at the end of the week when wearing accelerometre. April-June 2010	93.9% (n = 1266)

MTI Actigraph	Adolescents	Students wore an accelerometer (MTI Actigraph, GT3X) in 7 days. 5 week days and 2 weekend days	All day in 1 week (5 weekdays and 2 weekend days) in April-June 2010	88.6%* (n = 1194)

Questionnaire	Parents	One parent (mother or father) completed an e- survey about parental physical activity and health behaviour, the neighbourhood etc.	Duration: 15 minutes. In April-June 2010	72.6% (n = 978)

Questionnaire	School management	A person from the school management completed an e-survey about the school social and physical environment, school politic etc.	Duration: 10 minutes. In April-June 2010	100% (n = 14)

*Response rate calculated as those wearing accelerometer for at least 9 hours in at least 4 days (time interval 7a m-11 pm. cut out blocks of non wear defined as 20 min of inactivity - consecutive zeroes).

#### Pilot study

Prior to baseline (in February 2010), a comprehensive pilot study was conducted containing objective measurements of PA, questionnaires, diaries, and physical fitness and anthropometric tests. Focus group interviews with adolescents in 5-6^th ^grade and interviews with the school personnel at the pilot school were carried out to improve data collection procedures. Based on the experiences from the pilot study, smaller changes and adjustments were made.

### Baseline

The baseline measurements took place in April-June 2010. Data collection was conducted simultaneously at the intervention school and the paired control school. Three to four weeks before data collection, information materials were sent to the schools describing details, collection measures, and detailed timetables of the study. At every school, an employee was appointed as a contact person being responsible for: a) distributing of study materials to the students and involved teachers, b) collecting all diaries and accelerometers, c) booking of the school's computer lab for the questionnaire session, and d) reserving the school gym for physical fitness and anthropometric tests.

#### Questionnaires

Three different questionnaires were used - for students, parents, and school management, respectively. All questionnaires were internet based.

##### Student Questionnaires

The questionnaire for students contained a total of 48 questions on PA in leisure time, sedentary behaviour, active transport, health related behaviour, self-reported health status, living conditions, school satisfaction, and assessment of neighbourhood (physical environment, perception of safety, social capital etc.) and possibilities for being physical active in the neighbourhood and school setting. The majority of questions have previously been validated and/or used in other (inter)national surveys about children and adolescents' health (e.g. Health Behaviour in School-aged Children) [31]. The students completed the questionnaire on a computer in one school lesson (45 minutes) during the week wearing accelerometers prior to the physical fitness and anthropometric tests. A teacher was present during the session and instructed the students how to get started and handed out a unique password to each of the students to access the questionnaire. The teacher helped if there were any questions or problems with comprehension. It took 30-40 minutes to complete the questionnaire. All students were instructed to fill out the questionnaire individually, without talking to classmates. In June 2010, participating schools were requested to conduct a session for (the few) students who were not present at the first questionnaire session. This increased the already high response rate from 94.3% to 97.4% (table 1).

##### Parent Questionnaires

The questionnaire for parents contained questions on their own physical activity behaviours and their general overall health status as well as living conditions, assessment of neighbourhood (physical environment, perception of safety, social capital etc.) and the possibilities for being physically active in their neighbourhood. Only one parent for each student was requested to answer the questionnaire. Three to four weeks before the physical testing of students, parents received a unique password and a link to the questionnaire together with an information leaflet about the SPACE study. An estimated 10-15 minutes were projected to complete the questionnaire. In June 2010, a reminder was sent by the school intranet to those parents who did not initially fill out the questionnaire. This increased the response rate from 70.0% to 72.6% (table 1).

##### School Administrator Questionnaires

The questionnaire for school management included questions about the school's physical, socio-cultural, educational and political environment, with a special focus on initiatives regarding PA and general health of the students. The questionnaire was completed by the school principal or a similar administrative contact person. The questionnaire was filled out during the data collection period in April-June 2010, and took approximately 10 minutes to complete.

#### Student Assessment Materials

##### MTI Actigraph

Objective PA levels were obtained with the tri-axial Actigraph GT3X Activity Monitor [32]. The Actigraph detects acceleration in vertical, horizontal and transverse axes. All students who participated in the project were asked to wear an accelerometer for seven consecutive days on an elastic belt around the waist. Verbal and written instructions by trained professionals or by the research staff were given to the students regarding its use. All children were instructed to wear the monitor continuously during the day. The data was recorded at 2 second epoch, with total PA to be expressed in counts per minute. The monitor has been previously validated in both children and adolescents against a range of different outcomes [33-35]. To increase participation, the adolescents or one of their parents were offered a free text message reminder on their phone every morning.

##### Active commuting Diary

On weekdays when wearing the accelerometers, the students filled out a short diary during the first school lesson. The diary contained questions about transport to/from school and biking in general for the previous day. Furthermore at the end of the week, the students were asked about their use and reason for non-use of the accelerometer.

##### Physical fitness and anthropometric tests

The test lasted approximately 11/2 hours for each class and was conducted in the school gym or a nearby sports hall during school hours. A test team consisted of 5-6 people; a test leader, and 4-5 trained assistants. The contact person and/or the class teacher were present during the physical fitness and anthropometric tests and helped where needed. At the beginning of the session, all adolescents were introduced to the program. All participants in the test received written feedback with their own results on the below mentioned measures and tests.

*Anthropometry*. Height, weight, and waist circumference were assessed by standard anthropometric procedures. Height was measured with a portable stadiometer (SECA Leicester portable Height Measure). Students were asked to take off their shoes and stand with their back to the stadiometer. The sliding head piece of the stadiometer was lowered so that the hair was pressed flat, and height was recorded to the nearest millimetre.

Body weight was measured with a medical scale (Tanita BWB-800S Digital Scale). The participants were asked to take off shoes and pants if wearing long pants, e.g. jeans. The weight was recorded to the nearest decigram.

Waist circumference was measured by use of body tape (Chasmors WM02 Body Tape, Chasmors Ltd., London, UK). The participants were asked to stand with their feet fairly close together with the weight equally distributed on each leg, and to breathe normally. The reading of the measurement was taken at the end of exhalation. The waist was measured at the navel level with the tape wrapped around the body in a horizontal alignment. The waist circumference was recorded to the nearest 0.5 cm.

*Muscle strength *was measured by hand grip with Takei TKK A5401 Digital Handgrip Dynamometer. The test was performed with maximal exertion using the dominant hand with a straight arm at the side of the body. The test was repeated twice while conducting the agility test in between. The higher of the two measurements was recorded.

*Test of agility and speed*. The Eurofit test is a 10 × 5 m shuttle run that measures running speed and agility (ability to move quickly and change directions while maintaining control and balance). This test requires the person to run back and forth between the two parallel lines 5 m apart five times (50 m in total) as fast as possible. Both feet must fully cross the line every time [36].

*Aerobic fitness *was measured with the Andersen running test. Two parallel lines 20 m apart were made on the floor. Before the test start all participants were equipped with a Polar heart rate monitor and were thoroughly informed about the procedures. Eight to twelve subjects participated in the test at the same time. Subjects ran from one line to the other. They had to touch the line on the floor with one hand, turn around and run back. After 15 seconds, the test leader signalled the end of the test using a whistle and the subjects stopped immediately and rested for the next 15 seconds. This procedure was followed for 10 minutes. Subjects ran as fast as they could in order to cover the longest possible distance during the 10 minutes test run, and this distance was the test result [37].

*Isometric back muscle strength test *was measured by researchers in clinical biomechanics. Although not directly related to the SPACE study, these measures were also taken during physical fitness testing to determine associations between back muscle strength and other relevant health outcomes e.g. physical activity levels.

***Socio demographic and background variables *** were obtained from the questionnaires mentioned above, and from Statistics Denmark using the Danish Civil Registration System, which individually monitors information such as, gender, age, address, ethnicity, income and parental socioeconomic status for all residents of Denmark (each Danish citizen has a unique personal registration number).

### Outcome measures

**Figure 4:**
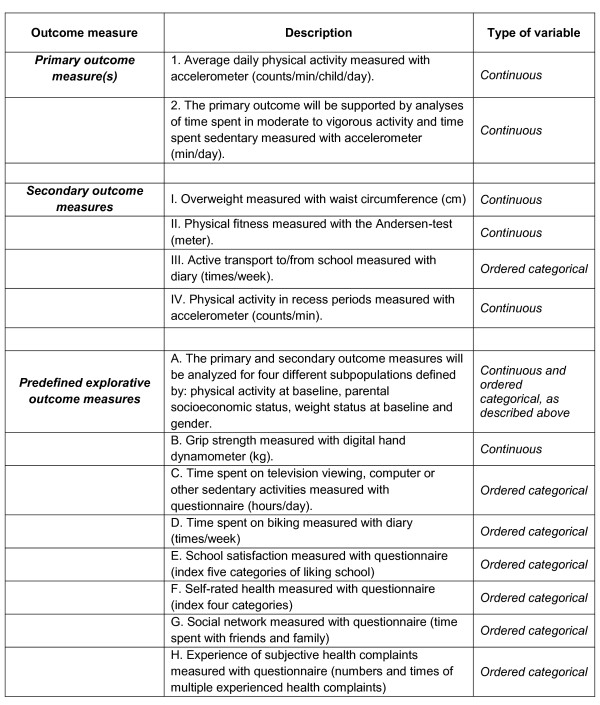
**Primary, secondary, and predefined explorative outcome measures**.

Primary, secondary, and predefined explorative outcome measures are registered at the Current Controlled Trials website [38]. An overview and description of the outcome measures is listed in Figure 4.

### Statistical analysis

The effect of the intervention will be tested using multilevel mixed effects models adjusted for relevant potential confounding factors (e.g. baseline outcome measure, gender, age, family socioeconomic status and parents' PA level). Because of the cluster structure of the data, random effects for school will be included in all analyses. In Figure 4 the primary, secondary and predefined explorative outcome measures are listed, together with the type of variable in each outcome measure. All primary and secondary outcome measures are continuous except for the secondary outcome measure no III regarding active transport to/from school, which is an ordered categorical variable that measures times of active transport during a week. The primary and secondary outcome measures are primarily based on assumptions of a normal distribution; however the secondary outcome measure about active transport is based on the assumption of a Poisson distribution. When more than one test is used, it is always important to be aware of the risk of type 1 error inflation. We have selected a primary outcome measure, that is deemed the most important, and then we have estimated 4 secondary distinct outcome measures to be acceptable given the effect size and quality of the study. Because of the risk of type 1 error, the results from the secondary outcome measures will be treated with caution.

The statistical software package, STATA (v.11.0, Texas, USA) will be used. The effectiveness of the intervention on adolescent physical activity will be assessed utilising an intention to treat approach [39].

## Discussion

A total of 1348 adolescents in 5-6^th ^grade in 56 classes in 14 schools in the Region of Southern Denmark participated in the baseline study (623 in intervention schools and 725 in control schools). In general, the response rate among students was high for all baseline measurements obtained from the questionnaire (97.4%), diaries (96.6%), physical fitness and anthropometric tests (93.9%), MTI Actigraph (min. 9 hr in 4d) (88.6%). A total of 978 parents responded to the parental survey (response rate: 72.6%), while all 14 school administrators filled out corresponding questionnaires (table 1). Table 2 presents baseline characteristics of the study population. Overall, 48.4% were girls, average age 12.5 years, BMI 18.9 kg/m^2^, height and weight 47.2 kg and 157.6 cm, waist circumference 69.8 cm, average meters covered in the fitness running test were 1000.3 meters, and average physical activity counts per minute measured by accelerometer were 609.3 counts/min. There were no significant differences between intervention (n = 623) and control (n = 725) groups at baseline according to above mentioned characteristics.

**Table 2:** Baseline participant characteristics

	Total Baseline population (n = 1194-1348)*	Intervention group (n = 574-623)*	Control group (n = 633-725)*	Difference between intervention and control (p-value)
*Gender, No. (%)*				
*Girls*	652 (48.4)	307(49.3)	345 (47.4)	38
*Boys*	696 (51.6)	316 (50.7)	380 (52.6)	64 (p = .54)

*Age, mean (SD), y*	12.5 (0.63)	12.4 (0.63)	12.5 (0.62)	0.05 (p = .17)

*BMI, mean (SD), kg/m^2^*	18.9 (3.01)	18.9 (3.00)	18.8 (3.03)	0.09 (p = .59)

*Weight, mean (SD), kg*	47.2 (10.18)	47.2 (10.04)	47.2 (10.31)	0.08 (p = .89)

*Height, mean (SD), cm*	157.6 (8.37)	157.5 (8.20)	157.7 (8.53)	0.22 (p = .65)

*Waist circumference*, *mean (SD), cm*	69.8 (8.77)	70.1 (8.83)	69.5 (8.71)	0.67 (p = .17)

*Physical fitness*, *mean (SD), meters*	1000.3 (103.47)	1000.6 (103.09)	1000.1 (103.89)	0.52 (p = .93)

*Physical activity*, *mean (SD), counts/min^#^*	609.3 (241.4)	596.7 (229.6)	620.3 (250.7)	19.4 (p = .09)

* n varies due to different sources of data. ^#^Mean counts/min calculated using only the vertical axis.

The purpose of this article was to give an overview of the study design in the SPACE Study as there is a growing demand for describing complex behaviour change interventions and to produce greater clarity about the functional components of those interventions [40]. In a review by Michie and colleagues, it was concluded that interventions were described in detail in only 5-30% of experimental studies [41].

Strengths of the SPACE study are the cluster randomized controlled study design and that it builds on a social ecological approach and thus acknowledges the importance of comprehensive multilevel interventions. Furthermore the study is conducted in a real life setting, where the usual staffs in municipalities and schools, under expert guidance from the research group, carries out the implementation of the intervention components. This also means that any findings of the trial are more generalisable because they are performed in the setting in which they are most likely to be implemented. This complies with the needs for more effectiveness trials, defined as interventions delivered under real-world conditions [42,43].

On the other hand, it is very important and a great challenge to ensure a certain degree of conformity of the implementation process when conducting effectiveness trials. We try to account for this issue in the study design, where process- and health economic analyses together with anthropologic observations will be carried out to address this issue concurrently with the effectiveness analysis. One relevant aspect with regards to this was that the financial crisis developed prior to the intervention, challenged fundraising for the built interventions considerably. Any financial difference will be examined in the future after the study is over. The implementation process is carefully followed to document what actually happens, and the municipal coordinators are responsible for conducting ongoing feedback and fill out evaluation forms of how the implementation process is carried out in the intervention schools, and to monitor if any extraordinary efforts in relation to physical activity and health are about to take place in the control schools.

We obtained a high response rate in the baseline measurements. Especially the measurements among adolescents conducted during the school day (questionnaire, diary, physical fitness and anthropometric tests) revealed a very high response rate (>93%). This demonstrates one of the strengths of conducting school based intervention research because the students had to be present in the school. Moreover an important explanation for the high response rate was the passive informed consent form used in the study. The school management and the contact person also played important roles by communicating information about the project and motivating students, parents, and other involved teachers to participate in the project. Even small differences between schools regarding engagement and commitment to the project are important as this can result in possible biases.

Physical activity is notoriously difficult to measure, and there are limitations of accelerometry as well as limitations in self-reported measurements of PA. We believe that the triangulation of methods used in this study will try to overcome measurement difficulties. A good example is that in Denmark, bicycling is an important mode of transportation but poorly registered by the accelerometer. Thus, measurement of cycling via self-reported measurements were applied. In our study, 78.4% of students in 5-6^th ^grade always or most of the time cycle to school according to the student questionnaire.

Finally it is worth to mention that we found no significant differences between the intervention and control group at baseline, suggesting that the matching with the audit tool was appropriate for creating equal groups.

## Conclusion

This manuscript provides a description of the study design, data collection, intervention components and the implementation of a multicomponent intervention study aiming at increasing physical activity levels of adolescents. The SPACE study is based on a social ecological framework, which is designed to implement organizational and structural changes in the school environment. In conclusion the SPACE Study is believed to contribute to new insights regarding multicomponent physical activity interventions in youth, and the results of the study may be used a basis for the development of further health enhancing PA planning in school aged adolescents.

## Competing interests

The authors declare that they have no competing interests.

## Authors' contributions

JT is the Principal Investigator of the SPACE study. MT led the writing for this manuscript with contributions and critical comments from the other three authors. MT and LBC were responsible for and carried out the quantitative data collection procedures in the SPACE study. All authors contributed to the research design, provided comments on the drafts, and read and approved the final manuscript.

## Pre-publication history

The pre-publication history for this paper can be accessed here:

http://www.biomedcentral.com/1471-2458/11/777/prepub
